# The natural atypical scrapie phenotype is preserved on experimental transmission and sub-passage in *PRNP *homologous sheep

**DOI:** 10.1186/1746-6148-6-14

**Published:** 2010-03-10

**Authors:** Marion M Simmons, Timm Konold, Lisa Thurston, Susan J Bellworthy, Melanie J Chaplin, S Jo Moore

**Affiliations:** 1 Department of Pathology, Veterinary Laboratories Agency Weybridge, New Haw, Addlestone KT15 3NB, UK; 2 Molecular Pathogenesis and Genetics Department, Veterinary Laboratories Agency Weybridge, New Haw, Addlestone KT15 3NB, UK

## Abstract

**Background:**

Atypical scrapie was first identified in Norwegian sheep in 1998 and has subsequently been identified in many countries. Retrospective studies have identified cases predating the initial identification of this form of scrapie, and epidemiological studies have indicated that it does not conform to the behaviour of an infectious disease, giving rise to the hypothesis that it represents spontaneous disease.

However, atypical scrapie isolates have been shown to be infectious experimentally, through intracerebral inoculation in transgenic mice and sheep. The first successful challenge of a sheep with 'field' atypical scrapie from an homologous donor sheep was reported in 2007.

**Results:**

This study demonstrates that atypical scrapie has distinct clinical, pathological and biochemical characteristics which are maintained on transmission and sub-passage, and which are distinct from other strains of transmissible spongiform encephalopathies in the same host genotype.

**Conclusions:**

Atypical scrapie is consistently transmissible within AHQ homozygous sheep, and the disease phenotype is preserved on sub-passage.

## Background

Atypical scrapie was first identified in Norwegian sheep in 1998 [1], and has subsequently been identified in many European countries [2] and elsewhere around the world, including North America [3] and the Falkland Islands [4]. Retrospective studies have identified cases predating this initial identification [5,6] and epidemiological studies have indicated that this form of scrapie does not conform to the behaviour of an infectious disease [7]. In many cases, atypical scrapie does not co-exist with classical scrapie (e.g. on individual farms, but also at a national level, such as in Portugal and the Falkland Islands [8]), although some field cases originate from flocks which have also reported classical scrapie, and it has occurred in a closed research flock with endemic classical disease [9]. It has also been reported in a research flock in which detailed biosecurity measures have been maintained, founded with sheep from a country free of classical scrapie [10]. This has led to proposals [2,10,11] that this form of scrapie is in fact a spontaneous disease which may have existed undetected for a long time.

Despite lack of evidence for infectivity in the epidemiological data, atypical scrapie isolates have been shown to be infectious experimentally, through intracerebral inoculation into ovinised transgenic mice [12] and sheep [13].

The interactions of host and infecting strain on ovine transmissible spongiform encephalopathies (TSEs) are known to be complex, and have a profound effect on the resulting phenotype of disease. Following experimental challenges distinct 'strain' characteristics can be identified [14], but when naturally-occurring disease is studied it is clear that host prion protein gene (*PRNP*) genotype can have a significant effect on the resulting phenotype [15], although a single genotype may support more than one phenotype [15,16]. The preferred genotype 'target' range of atypical scrapie is different to that of classical scrapie [17,18] but there is some overlap in susceptible genotypes.

In contrast to the widespread variation in pathology and immunopathology characteristics seen in classical scrapie [15], the pathology observed in atypical cases appears to be much more consistent, regardless of genotype [1,19-21].

The first successful challenge of an AHQ/AHQ sheep with 'field' atypical scrapie from an homologous donor sheep was reported in 2007 [13]. This paper provides data on further homologous AHQ/AHQ transmissions, including sub-passage of the initial case, and compares this with similar data from field cases of classical scrapie, and experimental bovine spongiform encephalopathy (BSE) in the same genotype.

## Results

Full details of all the animals presented in this paper are given in Table 1. In addition to the AHQ/AHQ animals intracerebrally challenged with atypical scrapie (cases 1-9), data is presented on the experimental classical scrapie positive control animals which were also intracerebrally challenged (cases 18-20).

**Table 1:** Individual case data of genotype, breed, inoculum, route of inoculation, incubation periods and clinical signs

Case no	Breed/genotype	Challenged with	Route	Age at challenge (months)	Ip (days)	Clinical signs
1*	Cheviot AHQ/AHQ	AHQ -atypical passive (PG1347/04) Portland	Intracerebral	5.5	378	Behavioural changes (confusion, separation from others), compulsive behaviour (spontaneous nibbling, circling clockwise), mild hind limb ataxia, weight loss, normal menace response, no signs of pruritus

2	Cheviot AHQ/AHQ	As above	Intracerebral	5.5	1057	Compulsive behaviour (circling anti-clockwise), absent menace response in the left eye, head tremor, general ataxia, no weight loss, no signs of pruritus

3	Cheviot AHQ/AHQ	Active surveillance - breed not known	Intracerebral	5.5	878	Behavioural change (nervousness), bilateral absent menace response, head tremor, general ataxia, minor weight loss

4	Cheviot AHQ/AHQ	Same case as above	Intracerebral	6	990	Behavioural changes (confusion, separation from others), bilateral absent menace response, head tremor, general ataxia, no weight loss, no signs of pruritus

5	Cheviot AHQ/AHQ	Subpassage of material from case 1	Intracerebral	3	667	No behavioural changes, bilateral absent menace response, general ataxia, no weight loss, no signs of pruritus

6	Cheviot AHQ/AHQ	Subpassage of material from case 1	Intracerebral	3	747	Compulsive behaviour (circling anti-clockwise), bilateral absent menace response, head tremor, general ataxia & hypermetria, minor weight loss, no signs of pruritus

7	Cheviot AHQ/AHQ	Subpassage of material from case 1	Intracerebral	3	671	Behavioural changes (confusion), bilateral absent menace response, head tremor, general ataxia, minor weight loss, no signs of pruritus

8	Cheviot AHQ/AHQ	(PG519/06) AHQ/AHQ passive case - Cheviot	Intracerebral	3	607	Behaviour changes (dullness, teeth grinding), normal menace response, head tremor, general ataxia, weight loss, no signs of pruritus

9	Cheviot AHQ/AHQ	(PG519/06) AHQ/AHQ passive case - Cheviot	Intracerebral	3	833	Behavioural changes (confusion, separation from others), compulsive behaviour (circling anti-clockwise), normal menace response, general ataxia, minor weight loss, no signs of pruritus

10	Cheviot AHQ/AHQ	Bovine BSE	oral	4	573	Abnormal behaviour (confusion), signs of pruritus (frequent rubbing, positive scratch test, wool loss), mild ataxia

11	Cheviot AHQ/AHQ	Bovine BSE	oral	4.5	595	Head tremor, signs of pruritus (frequent rubbing, wool loss, positive scratch test), minor weight loss

12	Cheviot AHQ/AHQ	Bovine BSE	oral	4.5	617	Signs of pruritus (wool loss), weight loss

13	Cheviot AHQ/AHQ	Bovine BSE	oral	8	642	Abnormal behaviour (separation from others), normal menace response, head tremor, signs or pruritus (frequent rubbing), mild ataxia, weight loss

14	Cheviot AHQ/AHQ	Bovine BSE	oral	4.5	664	Abnormal behaviour (teeth grinding), signs of pruritus (frequent rubbing, wool loss), weight loss

15	British milksheep X Finn Dorset AHQ/AHQ	Classical scrapie	Natural	Not applicable	9 years at clinical onset	Abnormal behaviour (teeth grinding), ataxia, no signs of pruritus

16	British milksheep X Finn Dorset AHQ/AHQ	Classical scrapie	Natural	Not applicable	5 years at clinical onset	No behavioural abnormalities, mild ataxia, pruritus (positive scratch test)

17	British milksheep X Finn Dorset AHQ/AHQ	Classical scrapie	Natural	Not applicable	3 years 9 months at clinical onset	No behavioural abnormalities, mild ataxia, signs of pruritus (frequent rubbing)

18	Cheviot ARQ/VRQ	Classical scrapie	Intracerebral	10	537	Abnormal behaviour (nervousness, separation from others), normal menace response, head & body tremor, signs of pruritus (positive scratch test, rubbing with nibble reflex), ataxia, weight loss

19	Dorset ARQ/VRQ	Classical scrapie	Intracerebral	10	605	Abnormal behaviour (nervousness), normal menace response, head tremor, signs of pruritus (rubbing with nibble reflex), mild ataxia, weight loss

20	Dorset VRQ/VRQ	Classical scrapie	Intracerebral	11	126	Abnormal behaviour (dullness), absent menace response in the eye, signs of pruritus (positive scratch test, frequent rubbing), hind limb ataxia, minor weight loss

Ip = incubation period.

* This case previously reported [13].

The scale of such studies, and the limited availability of both inoculum and recipient sheep precludes a study design which includes every genotype/strain/route of challenge combination. To enable us to assess the ability of a single *PRNP *genotype to support various TSEs with distinct pathological characteristics, data is presented for comparison from AHQ/AHQ animals orally challenged with BSE (cases 10-14) [22-24] and field cases of classical scrapie which have arisen naturally in AHQ/AHQ sheep (cases 15-17) [25,26].

Animals challenged with field atypical scrapie succumbed to disease from 378 - 1057 days post challenge (mean 751 days), which compares with 126, 537 and 605 days for the one VRQ/VRQ and two ARQ/VRQ classical scrapie intracerebrally inoculated controls respectively.

### Clinical

Full details of the clinical signs displayed by each animal are given in Table 1. Unlike classical scrapie and ovine BSE, atypical scrapie did not appear to present with any signs of pruritus. Ataxia was observed in all atypical scrapie cases. Four of nine atypical cases displayed signs of an asymmetric lesion distribution in the brain, such as circling to one side and a unilateral absent menace response. This was however not consistently displayed in all animals inoculated with the same material.

### Pathology and immunohistochemistry

Both the vacuolation profiles and immunopathological characteristics were very consistent within the animals challenged with atypical scrapie, and distinct from the other AHQ/AHQ animals where disease was attributable to other strains.

**Figure 1:**
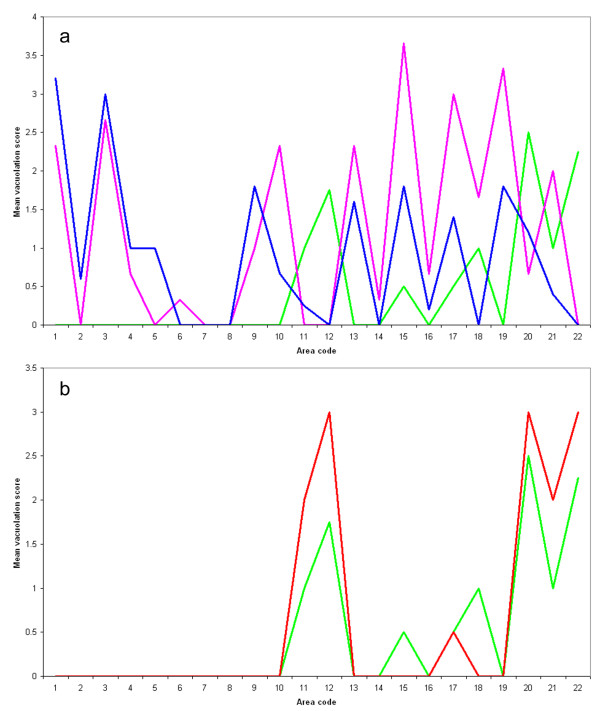
**Vacuolation profiles for atypical scrapie, classical scrapie and BSE in AHQ/AHQ sheep**. See Table 1 for details of the cases, and Table 3 for area code key, and mean ± SD for each data point. a) The profiles for the classical scrapie (pink, cases 15-17) and ovine BSE (blue, cases 10-14) cases are similar, particularly in terms of brainstem involvement, whereas the atypical scrapie profile (green, cases 1-9) has distinctive peaks in the cerebellum (area 12) and frontal cortex (area 20). b) The vacuolation profiles for experimental atypical scrapie cases subdivided by passage. The profile from the original challenged animals (green, cases 1-4 and 8-9) is very similar to that in the animals that succumbed to disease following sub-passage (red, cases 5-7).

Full vacuolation profiles could only be obtained for four of the five primary transmission cases, and one sub-passage animal. Vacuolation (Figures 1a, b) in the atypical cases was absent in the brainstem, and most pronounced in the cerebellar and cerebral cortices, as compared to the BSE and classical scrapie cases in which brainstem lesions were predominant. In the thalamic and frontal areas the main difference between the classical scrapie and BSE profiles related to the relative intensity of vacuolation, which was greater in the classical scrapie cases.

**Figure 2:**
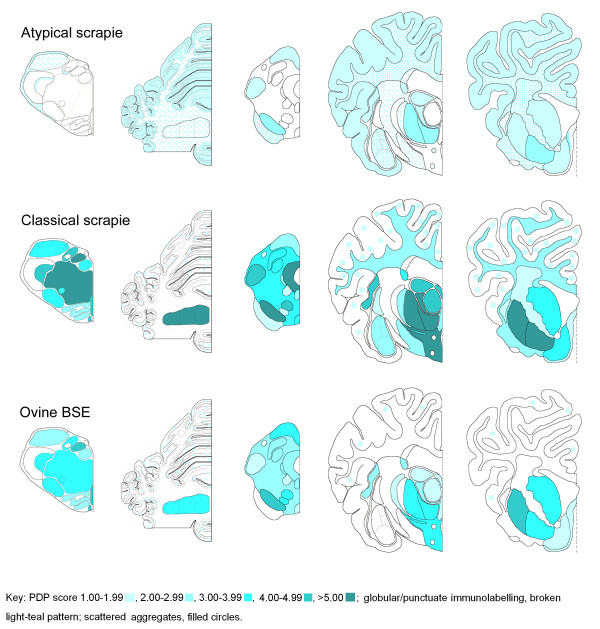
**PrP^Sc ^deposition pattern (PDP) maps for atypical scrapie, classical scrapie, and BSE in AHQ/AHQ sheep**. Note that, in comparison to classical scrapie and ovine BSE, the atypical scrapie cases have relatively sparse immunolabelling in the obex and rostral midbrain, widespread and prominent immunolabelling in the cerebellar cortex and white matter, sparse immunolabelling in the hypothalamus, and prominent immunolabelling in the neocortex and cortical white matter.

**Figure 3:**
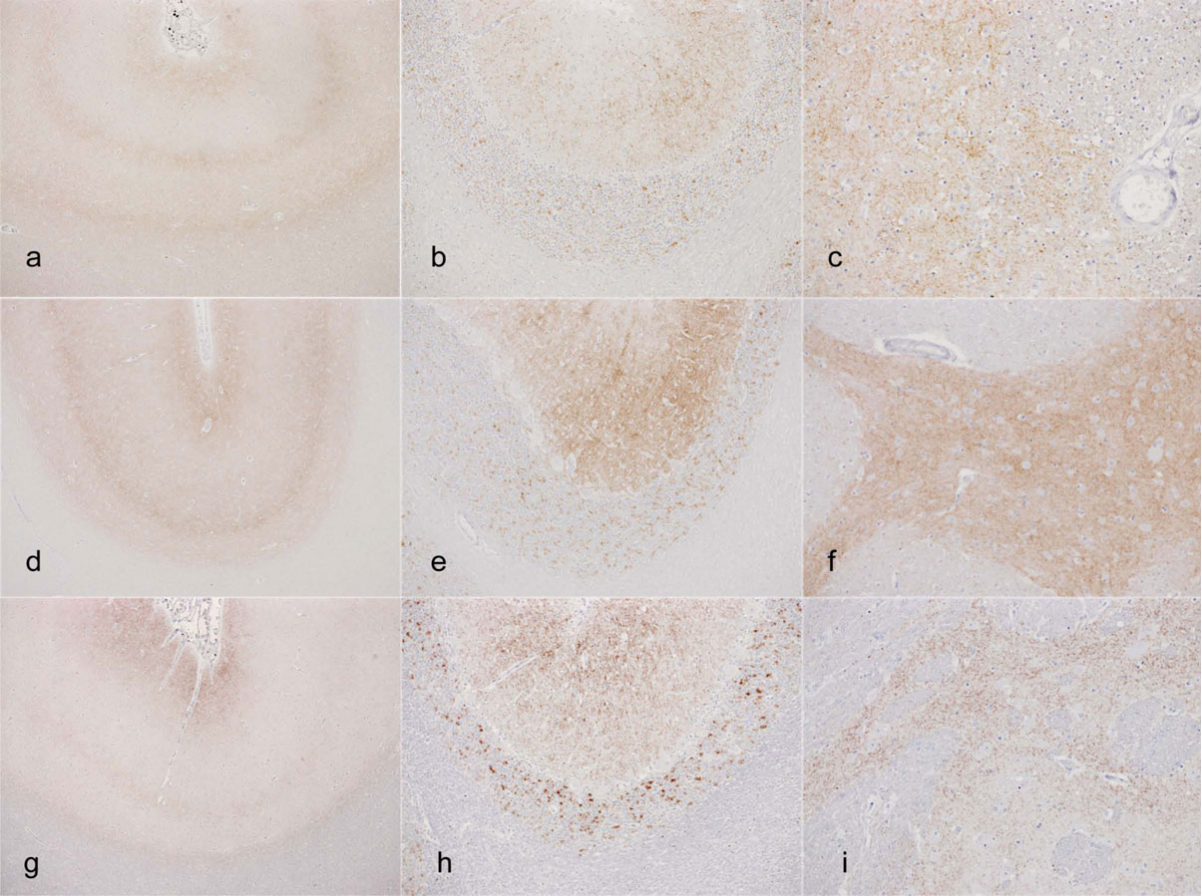
**Immunolabelling in atypical scrapie cases**. Immunolabelling characteristics in atypical scrapie are unchanged in natural disease (a-c), and primary (d-f) and secondary (g-i) passage. Note the characteristic three-band pattern in the neocortex (a, d, g); prominent immunolabelling in the molecular and granular cell layers of the cerebellum (b, e, h); prominent fine granular immunolabelling in the basal nuclei (c, f, i).

Immunohistochemistry (IHC; Figure 2) shows that immunolabelling patterns are also distinct, with both the type of labelling and the neuroanatomical distribution being different for the atypical scrapie compared to the other strains. However, the atypical scrapie pattern remains the same on primary versus secondary passage (Figure 3), and is consistent with the IHC patterns described in natural disease [20].

**Figure 4:**
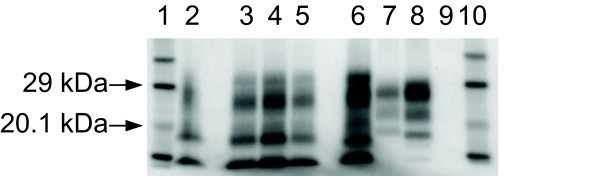
**Western immunoblot of caudal medulla of the atypical scrapie cases**. WB using monoclonal antibody Sha31, showing the consistency of blot characteristics between field case, experimental and sub-passaged atypical scrapie. Lanes 1 and 10 Biotinylated markers. Lane 2 Case 4. Lane 3 Case 8 (the sub-passage of Case 4). Lanes 4 and 5 Cases 7 and 5, both sub-passages of Case 1 (not shown). Lane 6 Field case atypical scrapie positive control. Lane 7 Field case classical scrapie control. Lane 8 Bovine BSE positive control. Lane 9 Scrapie negative control.

Western immunoblotting (WB; Figure 4) shows that the banding pattern associated with atypical scrapie [1,27] is present in all the atypical challenged cases, and is consistent regardless of whether it is primary or secondary passage (Figure 4). The blot of the field case which was examined in the context of a retrospective review of natural disease [28] was consistent with classical scrapie. The BSE cases were blotted within the remit of their source project (Chaplin and Bellworthy, unpublished data), and conform to the blot pattern (low molecular mass of the unglycosylated band with antibody 6H4 and a weak P4 antibody reaction) associated with experimental BSE in sheep.

No immunolabelling was seen in any lymphoid tissue from the atypical challenged sheep, despite widespread involvement of the equivalent tissues in the positive classical scrapie controls and the BSE-challenged sheep (Table 2).

**Table 2:** Immunolabelling results for lymphoid tissues from sheep challenged with experimental atypical or classical scrapie

Case number		1	2	3	4	5	18	19	20
Lateral retropharnyngeal lymph node		Neg	Neg	Neg	NA	Neg	Pos	Pos	Neg

Palatine tonsil		Neg	NA	NA	Neg	Neg	NA	NA	NA

Nictitating membrane		IT	IT	Neg	Neg	IT	Neg	IT	Neg

Spleen		Neg	Neg	Neg	Neg	Neg	Pos	Pos	Pos

Mesenteric lymph node		Neg	Neg	Neg	Neg	Neg	Pos	Pos	Pos

Distal ileum - Peyer's patches		Neg	Neg	Neg	Neg	Neg	Pos	Pos	Neg

Recto-anal mucosa associated lymphoid tissue (RAMALT)		Neg	Neg	Neg	Neg	Neg	Pos	Pos	Pos

Cases 1-5: atypical scrapie.

Cases 18-20: classical scrapie.

Neg = Negative; Pos = Positive; IT = insufficient lymphoid tissue to test; NA = sample not available.

## Discussion

This data confirms the experimental transmissibility of atypical scrapie, and the stability of disease phenotype - clinical, biochemical and pathological - on *PRNP *homologous experimental transmission in AHQ/AHQ animals. The mean incubation time of these cases (751 days) is approximately 50% of the average age at death in field cases with atypical scrapie [20]. This observation that intracerebral challenge shortens the incubation period is consistent with the substantial reductions in incubation period which are seen in the classical scrapie controls which are 90% (for VRQ/VRQ) and 60% (for VRQ/ARQ) less than the average age at onset of field cases (Ortiz-Pelaez, personal communication) in these genotypes.

There was a slight shortening of mean incubation period between the initial experimentally challenged sheep (790 days) and those which succumbed following sub-passage (695 days), but the group sizes are too small to draw any robust conclusions from this observation.

The wide range of incubation periods seen cannot be readily interpreted due to the very small numbers of animals in this study (a necessary restriction due to the very limited amount of suitable donor material, and limited number of recipients of a suitable genotype). It is interesting to note that the outliers (cases 1 and 2, Table 1) received the same inoculum. All donor inocula give similar incubation periods in ovinised transgenic (Tg338) mice when inoculated as part of a separate study (Spiropoulos, personal communication), although some difference in titre is likely to be masked by the sensitivity of these mice. In one titration of an atypical scrapie isolate the mean incubation period did not change more than 10% over the first three log dilutions (Spiropoulos and Simmons, unpublished data).

The absence of any visible lymphoreticular system (LRS) involvement in the experimentally challenged animals is consistent with what has been observed in natural cases of atypical scrapie [13] and cannot be attributed to the experimental route of exposure, since the classical scrapie controls challenged by the same route had widespread labelling of disease-associated PrP (PrP^Sc^) in the lymphoid tissues.

The clinical presentation of atypical scrapie cases was different to classical scrapie or BSE in sheep: atypical scrapie did not appear to cause evident pruritus, whereas the scrapie and BSE cases described here usually presented with pruritus. Compulsive behaviour, such as circling, was only observed in atypical scrapie, and an impaired menace response was considerably more frequent in atypical scrapie cases. The difference in the clinical picture does not appear to be confined to the genotype or the route of inoculation since similar signs in the absence of pruritus have been observed in naturally affected atypical scrapie cases of various genotypes [1,10,29], whereas pruritus is frequent in naturally affected classical scrapie cases [26] or sheep orally challenged or naturally infected with the BSE agent under experimental settings [24]. Ataxia with hypermetria, head tremor and an absent menace response, which were predominantly observed in sheep with atypical scrapie, are signs indicative of a dysfunction of the cerebellum [30]. This concurs with the lesion and PrP^Sc ^immunolabelling pattern distribution in atypical scrapie, which is particularly prominent in the cerebellum. However, the lateralisation of clinical signs (circling to one side or a unilaterally deficient menace response) seen in atypical scrapie is unusual and to the authors' knowledge has not been described elsewhere in ovine BSE or classical scrapie. It is unlikely that the intracerebral route was responsible for the apparent lateralisation of clinical signs because it was not observed in ovine cases of BSE [24] and classical scrapie inoculated by the same route (cases 18-20, Table 1). Compulsive circling in the absence of vestibular signs like head tilt and nystagmus is suggestive of an asymmetric lesion of the forebrain, usually on the same side as the circling direction [31]. A unilateral absent menace response has been associated with a unilateral cerebellar lesion on the same side as the deficit [32]. This would suggest that the signs in case 2 (Table 1), which presented with a left absent menace response and circling to the left, was predominantly caused by a lesion of the left side of the brain. Unfortunately, the whole brain of this case was not available to evaluate symmetry of neuropathological changes, but there was no evidence of any pronounced asymmetrical lesion or PrP^Sc ^distribution in the bilateral sections examined in case 6, which also circled to the left.

## Conclusions

This study shows that a single *PRNP *genotype, AHQ/AHQ can display a range of disease phenotypes which are consistent and readily distinguishable from each other, supporting the suggestion that such characteristics can be attributable, predominantly, to the agent strain. However, the relative over-representation of the AHQ genotype in the atypical naturally-affected populations supports the hypothesis that field strains have strong genotype 'tropisms' or that there is genotype selection of strains, as proposed by Spiropoulos [15].

The consistent disease phenotype seen in natural disease, primary experimental transmission and subsequent passage, supports the experimental inoculation model as a valid tool for the study of atypical scrapie, regardless of whether the disease in the field is acquired or spontaneous. It also enables the creation of atypical scrapie samples in a consistent manner for test evaluation and/or quality assurance purposes, helping to improve surveillance approaches for disease detection and confirmation in the field.

## Methods

### Animals

Full details of all the animals used in this study are given in Table 1.

Six of the cases challenged with atypical scrapie (cases 1-6, Table 1) form part of a larger transmission study which has been described elsewhere [13] in which various homologous and cross-genotype challenges have been initiated by both the oral and intracerebral route. The other three challenges (cases 7-9, Table 1) were undertaken for the principal purpose of tissue production for use in test evaluation and quality assurance purposes for the European Community Reference Laboratory for TSE, and only brainstem, cerebellum and basal nuclei/frontal cortex were collected into fixative for the purpose of confirming disease.

In total, nine homologous AHQ/AHQ to AHQ/AHQ intracerebral challenges have been performed as part of a larger study, one of which has already been reported [13] and three of which (cases 5-7) were sub-passages of this first case. All of these, including the three sub-passage animals, have now succumbed to clinical disease. Three positive controls were challenged by the same method using field classical scrapie cases derived from the active surveillance programme (cases 18-20, Table 1). All recipient animals were sourced from the Veterinary Laboratories Agency's (VLA) New Zealand-derived flock [10].

All intracerebral inoculations were carried out under general anaesthesia, and in accordance with the United Kingdom (UK) Animal (Scientific Procedures) Act 1986, under Licence from the UK Government Home Office (Project licence no: 70/5780). Such licence is only granted following approval by the internal VLA ethical review process as mandated by the Home Office.

For the purpose of generating equivalent data from end-stage classical scrapie in the same *PRNP *genotype, three historical cases of classical scrapie in AHQ/AHQ sheep from a single flock (cases 15-17, Table 1), which presented through passive surveillance [25,26], were identified through a database query and sectioned, stained and profiled in the same way as the atypical cases.

Similarly, five cases of experimental BSE in sheep (orally challenged [22-24], cases 10-14, Table 1) were available to us for comparison, and sections were similarly prepared and scored.

### Clinical monitoring

A neurological examination was conducted when animal husbandry staff suspected clinical disease (for more details, see [13]).

### Pathology and immunohistochemistry

Whole brain was removed from each animal and hemisected longitudinally. One half of the brain was placed into 10% formal saline for histology, and the other half stored at -80°C. Prompted by the clinical observations in one case (case 6, Table 1) an additional section of cerebellum and cerebrum of the 'fresh' half of the brain was taken and fixed for comparison of lesion intensity and distribution with the other half of the brain. A range of lymphoid tissues were also collected from the experimental group.

All brain tissue was routinely fixed, processed into paraffin wax, sectioned and stained with haematoxylin and eosin as described in detail elsewhere [16]. Full details of the areas profiled are given in Table 3.

**Table 3:** Vacuolation scores for each neuroanatomical area in each group of AHQ/AHQ cases

		Mean vacuolation score (± SD)			
**Area Code**	**Neuroanatomical area**	**Atypical Primary challenge**	**Atypical Sub-passage**	**Classical scrapie**	**Ovine BSE**

		**(n = 4)**	**(n = 2)**	**(n = 3)**	**(n = 5)**

1	Dorsal nucleus of the vagus	0.00 (± 0.00)	0.00 (± 0.00)	2.33 (± 0.58)	3.20 (± 0.84)

2	Hypoglossal nucleus	0.00 (± 0.00)	0.00 (± 0.00)	0.00 (± 0.00)	0.60 (± 0.55)

3	Reticular formation	0.00 (± 0.00)	0.00 (± 0.00)	2.66 (± 0.58)	3.00 (± 0.71)

4	Midline raphe (caudal)	0.00 (± 0.00)	0.00 (± 0.00)	0.66 (± 0.58)	1.00 (± 0.82)

5	Accessory cuneate nucleus	0.00 (± 0.00)	0.00 (± 0.00)	0.00 (± 0.00)	1.00 (± 0.71)

6	Olivary nuclei	0.00 (± 0.00)	0.00 (± 0.00)	0.33 (± 0.58)	0.00 (± 0.00)

7	Vestibular complex	0.00 (± 0.00)	0.00 (± 0.00)	0.00 (± 0.00)	0.00 (± 0.00)

8	Cochlear nucleus	0.00 (± 0.00)	0.00 (± 0.00)	0.00 (± 0.00)	0.00 (± 0.00)

9	Nucleus of the spinal tract of the trigeminal nerve	0.00 (± 0.00)	0.00 (± 0.00)	1.00 (± 1.00)	1.80 (± 0.84)

10	Midline raphe (rostral)	0.00 (± 0.00)	0.00 (± 0.00)	2.33 (± 0.58)	0.67 (± 0.58)

11	Cerebellar vermis - nodulus	1.00 (± 0.82)	2.00 (± 1.41)	0.00 (± 0.00)	0.25 (± 0.50)

12	Cerebellar vermis -excluding nodulus	1.75 (± 1.26)	3.00 (± 0.00)	0.00 (± 0.00)	0.00 (± 0.00)

13	Central grey matter	0.00 (± 0.00)	0.00 (± 0.00)	2.33 (± 0.58)	1.60 (± 0.55)

14	Red nucleus	0.00 (± 0.00)	0.00 (± 0.00)	0.33 (± 0.58)	0.00 (± 0.00)

15	Substantia nigra	0.50 (± 0.58)	0.00 (± 0.00)	3.66 (± 0.58)	1.80 (± 1.30)

16	Lateral geniculate nucleus	0.00 (± 0.00)	0.00 (± 0.00)	0.66 (± 0.58)	0.20 (± 0.45)

17	Dorsomedial thalamic nuclei	0.05 (± 0.58)	0.50 (± 0.71)	3.00 (± 0.00)	1.40 (± 1.14)

18	Ventrolateral thalamic nuclei	1.00 (± 0.00)	0.00 (± 0.00)	1.66 (± 0.58)	0.00 (± 0.00)

19	Hypothalamus	0.00 (± 0.00)	0.00 (± 0.00)	3.33 (± 0.58)	1.80 (± 0.84)

20	Head of the caudate nucleus	2.50 (± 1.00)	3.00 (± 0.00)	0.66 (± 1.15)	1.20 (± 0.84)

21	Nucleus accumbens	1.00 (± 0.82)	2.00 (± 1.41)	2.00 (± 2.00)	0.40 (± 0.55)

22	Frontal cortex	2.25 (± 0.96)	3.00 (± 1.41)	0.00 (± 0.00)	0.00 (± 0.00)

See also Figure 1.

Immunohistochemical detection of PrP^Sc ^was performed using mouse monoclonal antibody 2G11 (Institut Pourquier, Montpellier, France), raised against ovine PrP peptide sequence 146-R154 R171-182.

Tissue sections were de-waxed and rehydrated routinely. Epitope demasking was performed by immersion of sections for 30 minutes in undiluted formic acid, then washed in running tap water for 15 minutes, followed by autoclaving at 121°C in citrate buffer pH 6.1 (8.8 mM tri-sodium citrate dihydrate, 1.3 mM citric acid in 2 litres purified water). Endogenous peroxidase was blocked using 3% hydrogen peroxide (100 vol) in methanol, and washing buffer used throughout the procedure was tris buffered saline, supplemented with 0.2% tween20 (TBST). Primary antibody was applied at dilution of 1/400 for 1 hour at room temperature, with immunodetection performed using biotinylated goat anti mouse and avidin-biotin-peroxidase-complex (Vector Elite, Burlingame, USA) technique using diaminobenzidine chromogen prepared in McIlvane's citrate buffer. Sections were counterstained using Mayer's haematoxylin, then routinely dehydrated, cleared and mounted in dibutylphthalate in xylene (DPX), before examination by light microscopy.

Vacuolation and immunohistochemistry profiles were created using standard subjective methods as previously described [16,20] in which the severity of vacuolar lesions, or the type of PrP immunolabelling, is assessed in a standard range of precise neuroanatomical areas. Some modifications were made to the original method [20] to accommodate the range of morphological PrP^Sc ^immunolabelling types seen across the three TSE strains. Globular and punctuate labelling types, which have only been observed in atypical scrapie cases [2,20,33], were not included in the calculation of the average scores for each area. Therefore average PrP deposition pattern scores (0 = negative to 6 = strong positive) for each area were calculated using only the remaining 12 immunolabelling types (intraneuronal, intraglial, intra-astrocytic, (fine) granular, stellate, linear, perineuronal, plaque-like, subpial, (sub)ependymal, perivascular, vascular plaques).

Tissues from the LRS of each challenged animal were examined by IHC, using the same method described above. The effects of the intracerebral route of challenge were controlled for by examination of the LRS from positive control VRQ/VRQ and ARQ/VRQ animals that had been challenged intracerebrally with classical scrapie (cases 18-20, Table 1).

### Western Immunoblot

Fresh brain samples were subjected to the TeSeE Universal WB (Bio-Rad Cat No: 355 1169 Marnes-la-Coquette, France).

A 0.35 g tissue sample from each case was ribolysed, purified, Proteinase K treated and PrP^Sc ^concentrated following the kit instructions and reagents supplied. Samples were heated for 4 minutes at 100°C and15 μl of each sample was loaded in single lanes onto pre-cast 12% bis-tris gels (Criterion, Bio-Rad) and electrophoresed for 50 minutes at 200 V. The proteins were then transferred onto PVDF membranes (115 V for 60 minutes) and blocked (Bio-Rad blocking solution) for 40 minutes at room temperature. They were probed with the kit primary antibody for 30 minutes at room temperature.

The membranes were washed, incubated for 20 minutes in Bio-Rad secondary antibody at room temperature, washed again and the membranes were incubated with ECL substrate (GE Healthcare, Amersham, UK) for 45-60 seconds. The signal was detected with the Fluor-S MultiImager (Bio-Rad).

Molecular mass markers were included at either end of the gel. A single lane each of a known UK classical scrapie, known UK bovine BSE and a known UK atypical scrapie case were included for profile comparisons.

Frozen material for the WB component of the study was only available from the experimental challenges in the current study. One of the scrapie cases was blotted as part of the retrospective study previously reported [28]. The other two cases were sampled before WB was a routine approach for TSE diagnosis, so no blot data is available from these animals.

All five ovine BSE cases were blotted using the VLA-Hybrid Western method [28].

## Authors' contributions

MMS led the project, analysed the data and drafted the manuscript. TK performed the clinical examinations and drafted the manuscript. LT led the technical support team and SJB managed the project which provided the BSE data. MJC performed the Western immunoblotting. SJM performed the IHC mapping and drafted the manuscript. All authors read and approved the final manuscript.
